# An Updated Meta-Analysis of Risk of Multiple Sclerosis following Infectious Mononucleosis

**DOI:** 10.1371/journal.pone.0012496

**Published:** 2010-09-01

**Authors:** Adam E. Handel, Alexander J. Williamson, Giulio Disanto, Lahiru Handunnetthi, Gavin Giovannoni, Sreeram V. Ramagopalan

**Affiliations:** 1 Wellcome Trust Centre for Human Genetics, University of Oxford, Oxford, United Kingdom; 2 Department of Clinical Neurology, University of Oxford, Oxford, United Kingdom; 3 Department of Physical and Theoretical Chemistry, University of Oxford, Oxford, United Kingdom; 4 Blizard Institute of Cell and Molecular Science, Barts and The London School of Medicine and Dentistry, Queen Mary University of London, London, United Kingdom; National Institutes of Health, United States of America

## Abstract

**Background:**

Multiple sclerosis (MS) appears to develop in genetically susceptible individuals as a result of environmental exposures. Epstein-Barr virus (EBV) infection is an almost universal finding among individuals with MS. Symptomatic EBV infection as manifested by infectious mononucleosis (IM) has been shown in a previous meta-analysis to be associated with the risk of MS, however a number of much larger studies have since been published.

**Methods/Principal Findings:**

We performed a Medline search to identify articles published since the original meta-analysis investigating MS risk following IM. A total of 18 articles were included in this study, including 19390 MS patients and 16007 controls. We calculated the relative risk of MS following IM using a generic inverse variance with random effects model. This showed that the risk of MS was strongly associated with IM (relative risk (RR) 2.17; 95% confidence interval 1.97–2.39; p<10^−54^).

**Discussion:**

Our results establish firmly that a history of infectious mononucleosis significantly increases the risk of multiple sclerosis. Future work should focus on the mechanism of this association and interaction with other risk factors.

## Introduction

Multiple sclerosis (MS) is a complex neurological disorder characterised by demyelination and axonal loss[1]. The relative contribution of genetic and environmental factors to MS aetiology is an area of fertile debate. It is becoming increasingly clear that a multitude of genetic and environmental factors interact at different points during the course of an individual's life to determine disease susceptibility[2]. These factors include the Human Leukocyte Antigen (HLA) region, Epstein-Barr virus (EBV), vitamin D and smoking[3], [4], [5].

The evidence implicating EBV in MS aetiology is growing[6]. MS patients are almost universally seropositive for EBV infection (99.5%) although there is a very high rate of asymptomatic EBV infection amongst control populations too (94.2%)[7], [8]. Furthermore, high titres of antibodies against EBV have been shown to predict conversion from the initial demyelination seen in a clinically isolated syndrome (CIS) to definite MS[9]. Elevated titres of antibodies directed against the latent phase antigen of EBV infection are also correlated with disease activity on MRI[10]. Finally, studies have suggested that a latitude gradient exists for the incidence of IM, resembling that of MS[11], [12].

EBV infection can occur either asymptomatically or present as infectious mononucleosis (IM). A previous meta-analysis of cohort and case-control studies have suggested that IM more than doubles the risk of MS (relative risk (RR) 2.3; 95% confidence interval (95% CI) 1.7–3.0; p<10^−8^) [13]. Given the high rates of asymptomatic EBV infection present both in patient and control populations, this implicates IM as the manifestation of EBV infection most associated with the development of MS. Since the original meta-analysis of IM and MS was published several other much larger studies have been directed towards understanding the association between IM and MS. Establishing the precise magnitude of the relationship between IM and MS is important in understanding the role of EBV in MS and also for attempts at risk prediction[14]. Therefore in the present study we aimed to broaden the previous meta-analysis to include studies published since 2006 to confirm the association of IM and MS and to see if the relationship between IM and MS is latitude dependent.

## Methods

### Search Strategy

We searched Medline from January 2006 until April 2010 for articles with the phrase: “(Epstein-Barr virus OR EBV OR human herpesvirus 4 OR HHV-4 OR infectious mononucleosis OR glandular fever) AND (multiple sclerosis OR MS OR disseminated sclerosis)”. This was the same strategy used by Thacker and colleagues in 2006[13]. We hand-searched abstracts generated from this search term for cohort or case-control studies and examined references of these articles for potential additional studies. Experts in the field were unaware of any ongoing unpublished studies for inclusion.

### Statistical analysis

We used the generic inverse variance with random effects model in Reference Manager 5.0 to calculate the overall RR, 95% CI and test statistic for the interaction and heterogeneity of studies. RRs were subsequently calculated for subgroups of studies and compared between case-control and cohort studies, and studies using definite MS diagnostic criteria and those using definite/probable MS. We extracted latitude from papers based on either the latitude of the study centre if performed in a distinct region or the geographical midpoint of the country if a national study. We tested for an interaction between latitude and sex-ratio by both a comparison of RRs based on the median value for each factor and also performed linear weighted fit on the natural log of RRs in Mathematica 7.0.1.0.

## Results

### Included studies

Our search criteria produced 150 studies. We identified four studies (one population cohort study and three case-control studies) not included in the original meta-analysis, making a total of 18 studies[15], [16], [17], [18], [19], [20], [21], [22], [23], [24], [25], [26], [27], [28], [29], [30], [31], [32]. The details of the studies included are shown in **table S1**. In terms of case-control studies, the new studies increased the number of MS cases included in the meta-analysis from 1642 to 19390 and controls from 3596 to 16007. The RR in all studies had a range of 0.8–17 and a median RR of 2.21.

### Risk of MS following IM

**Figure 1:**
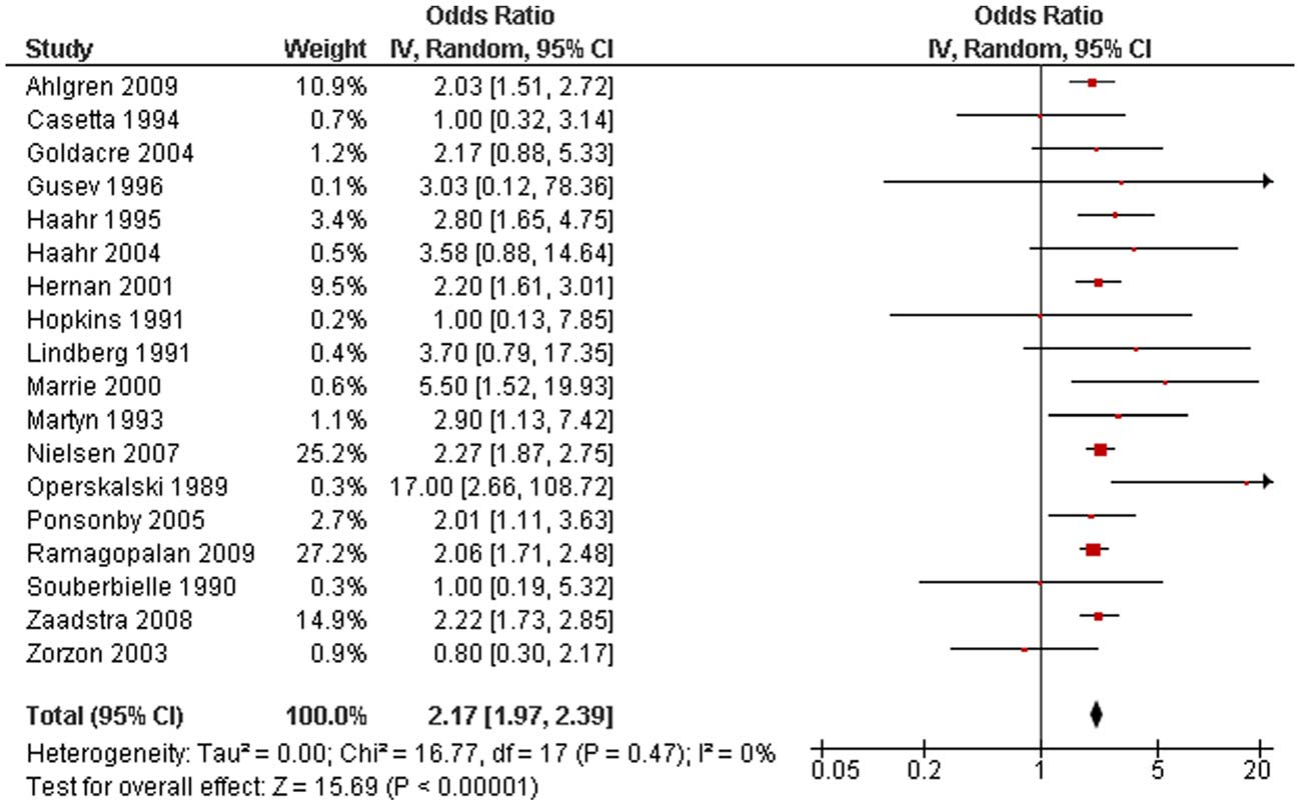
Forest plot of multiple sclerosis risk after infectious mononucleosis.

Including all of the 18 identified studies in our meta-analysis, we calculated a RR of 2.17 (95% CI 1.97–2.39; p<10^−54^; **figure 1**). There was no significant heterogeneity between studies (p = 0.47). There were no significant differences detected when comparing subgroups of studies based on design or diagnostic criteria, or when dichotomised based on median latitude or sex-ratio (**table 1**).

**Table 1:** Relative risk and 95% confidence intervals for different subgroups of studies.

Subgroup of studies	Relative risk	Lower 95% CI	Upper 95% CI	number of studies	p-value for comparison
Case-control	2.11	1.82	2.43	14	0.39
Cohort	2.33	1.96	2.78	4	
Definite MS	2.12	1.69	2.64	5	0.74
Definite/probable MS	2.21	1.97	2.47	10	
Latitude <54	2.01	1.51	2.67	9	0.56
Latitude ≥54	2.2	1.96	2.47	9	
Sex-ratio <2.12	2.03	1.54	2.67	7	0.61
Sex-ratio ≥2.12	2.21	1.85	2.63	8	

P-values for comparison between subgroups are also shown.

### Interaction with latitude and sex-ratio

**Figure 2:**
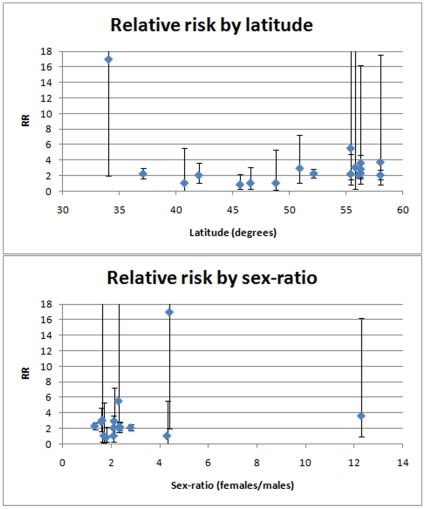
Plots of relative risk against latitude and sex-ratio. Error bars show 95% confidence intervals of relative risk estimates.

We tested both latitude and sex-ratio for correlation with the RR for each study using linear weighted fit (**figure 2**). No significant correlation was found for either (latitude: r^2^<0.01, p = 0.91; sex-ratio: r^2^<0.01, p = 0.79).

## Discussion

With an MS patient sample size an order of magnitude larger than the previous meta-analysis, our study has established unequivocally that IM is a significant risk factor for the development of MS. The robust nature of this relationship is unsurprising given the wealth of serological research implicating EBV in MS aetiology. Meta-analysis has some limitations: notably publication bias of included studies and heterogeneity of study designs[33]. We have attempted to avoid these pitfalls in this study.

There was no strong evidence to suggest that the degree to which IM influences MS susceptibility varies by latitude, however the range of latitudes at which studies have been conducted is relatively small. This study cannot exclude an effect of EBV infection on MS latitudinal risk, as studies conducted so far include predominantly Caucasian cases and controls or are studies of IM and MS in predominantly Caucasian populations. Studies on other populations also afflicted by MS would be valuable in examining whether IM is a risk factor of equal magnitude in all populations, especially since EBV is known to interact with certain *HLA* alleles that vary in frequency between different populations[4]. MS susceptibility is defined by an interplay of many different genetic and environment risk factors and so it is likely that these all act in concert at different points in the disease process to establish the well-described latitudinal gradient[34]. Thus it is unsurprising that, even if IM has an important role in establishing MS prevalence patterns, examining this risk factor alone did not reveal any strong effects of IM in the latitudinal distribution of MS. It is unsurprising that we detected no interaction between IM and the sex-ratio of MS cases since there were no significant difference between the rates of IM in males and females in a recent cohort study[35].

The mechanism by which EBV infection is associated with MS is currently poorly understood. One hypothesis is that infection of any sort is an epiphenomenon in the development of MS, reflecting a generally increased propensity to infection or generally altered immune responses directed against infectious antigens[36], [37], [38]. Certainly some studies have cast doubt upon the specific role of current EBV infection in MS[39], although there is little doubt that past EBV infection is an important risk factor[8]. An alternative is that a lack of exposure to infectious agents early in life results in a failure of tolerance in the immune system: the “hygiene hypothesis”[12]. This certainly fits with an increased rate of IM in MS patients, since EBV infection later in life is more likely to result in IM. However, it is difficult to reconcile this with elevated MS risk in migrants moving from regions of low MS prevalence to high prevalence[40]. Also, whereas one would expect this to predict a difference in MS risk with birth order of siblings (due to differing exposure to infectious antigens), this was not observed in a large Canadian study[41].

IM has an important role in MS susceptibility but future work needs to examine how IM rather than asymptomatic EBV infection appears to be the main association with MS. The role of EBV in MS remains to be elucidated and furthermore, of increasing interest is attempts to understand how MS risk factors interact that lead to the development of MS[2], [8]. For this reason, prospective studies should try to investigate the reasons why some individuals present with IM after infection and why some of these individuals then develop MS.

## Supporting Information

Table S1 Characteristics of studies included in the meta-analysis.
(0.05 MB DOC)
