# Supplementary material for: An Updated Meta-Analysis of Risk of Multiple Sclerosis following Infectious Mononucleosis
Source: PLoS One. 2010 Sep 1;5(9):e12496. doi: 10.1371/journal.pone.0012496 (PMC2931696; doi:10.1371/journal.pone.0012496)
Supplement: Table S1 — Characteristics of studies included in the meta-analysis. (0.05 MB DOC) [file pone.0012496.s001.doc]

| **First author & year of publication** | **Type** | **Diagnostic criteria** | **Cases** | **Controls** | **Risk ratio** | **Lower 95% CI** | **Upper 95% CI** | **Female/male ratio** | **Case source** | **Control source** | **Ascertainment** |
| --- | --- | --- | --- | --- | --- | --- | --- | --- | --- | --- | --- |
| Operskalski 1989 | Case-control | Definite/probable MS | 145 | 145 | 17 | 2 | 81.8 | 4.4 | MS cohort (prevalent) | Friends | Interview/questionnaire |
| Souberbielle 1990 | Case-control | Definite MS Poser criteria | 153 | 153 | 1 | 0.19 | 5.37 | 1.7 | Hospital (incident) | Hospital | Interview |
| Hopkins 1991 | Case-control | Definite/probable MS Poser criteria | 16 | 61 | 1 | 0.09 | 5.55 | 4.3 | Community (prevalent) | Community | Interview |
| Martyn 1993 | Case-control | Definite MS, optic neuritis, isolated demyelination | 214 | 160 | 2.9 | 1.1 | 7.2 | 2.12 | Hospital (prevalent) | Hospital & blood donors | Interview |
| Casetta 1994 | Case-control | Definite MS McAlpine criteria | 104 | 150 | 1 | 0.31 | 3.06 | 2.1 | Community (prevalent) | Hospital & community | Interview |
| Gusev 1996 | Case-control | Definite/probable MS McAlpine criteria | 155 | 155 | 3.03 | 0.24 | 160.55 | 1.63 | Community (incident & prevalent) | Hospital & hospital staff | Interview |
| Marrie 2000 | Case-control | Definite/probable MS | 225 | 900 | 5.5 | 1.5 | 19.7 | 2.3 | Community (incident) | Community | Medical record |
| Hernan 2001 | Case-control | Definite/probable MS Poser criteria | 301 | 1416 | 2.2 | 1.6 | 3 | All female | Cohort (incident & prevalent) | Cohort | Questionnaire |
| Zorzon 2003 | Case-control | MS McDonald criteria | 140 | 131 | 0.8 | 0.3 | 2.2 | 1.8 | MS centre (prevalent) | Blood donors | Interview |
| Haahr 2004 | Case-control | Neurologist diagnosed MS | 53 | 53 | 3.58 | 0.97 | 16.23 | 12.3 | MS society (incident) | Friends | Questionnaire |
| Ponsonby 2005 | Case-control | Definite MS Poser criteria & imaging | 136 | 272 | 2.01 | 1.11 | 3.62 | 2.1 | MS society & neurologists (prevalent) | Voter rolls | Interview/questionnaire |
| Zaadstra 2008 | Case-control | Definite MS | 2877 | 2673 | 2.22 | 1.73 | 2.86 | 2.35 | MS registry | Randomly chosen from phone records | Questionnaire |
| Ramagopalan 2009 | Case-control | Definite/probable MS | 14362 | 7671 | 2.06 | 1.71 | 2.48 | 2.8 | Cohort (incident & prevalent) | Spouses | Questionnaire |
| Ahlgren 2009 | Case-control | Definite/probable MS & CIS McDonald/Poser criteria | 509 | 2067 | 2.03 | 1.52 | 2.73 | 2.35 | Cohort (prevalent) | Randomly chosen from population register | Questionnaire & health records |
| Lindberg 1991 | Cohort | Definite MS Poser criteria or laboratory results | 3 | N/A | 3.7 | 0.8 | 17.6 | Not reported | Hospital (incident) | Regional population | Medical record |
| Haahr 1995 | Cohort | Definite/probable MS Allison criteria & laboratory results | 16 | N/A | 2.8 | 1.6 | 4.6 | 1.6 | MS registry | National population | Heterophile antibody test |
| Goldacre 2004 | Cohort | Hospital admission for MS | 6 | N/A | 2.17 | 0.79 | 4.77 | Not reported | Hospital records (incident) | Hospital records | Medical record |
| Nielsen 2007 | Cohort | Definite/probable MS Allison criteria & laboratory results | 104 | N/A | 2.27 | 1.87 | 2.75 | 1.32 | MS registry | National population | Heterophile antibody test |
